# Artificial intelligence in scientific writing

**DOI:** 10.1590/1516-3180.2024.1425.26062024

**Published:** 2024-09-02

**Authors:** Isabele Alves Chirichela, Alessandro Wasum Mariani, Paulo Manuel Pêgo-Fernandes

**Affiliations:** I Fellow Department of Thoracic Surgery, Hospital das Clínicas (HCFMUSP), Faculdade de Medicina, Universidade de São Paulo (USP), São Paulo (SP), Brazil.; II Department of Thoracic Surgery, Hospital das Clínicas (HCFMUSP), Faculdade de Medicina, Universidade de São Paulo (USP), São Paulo (SP), Brazil.; III Vice-director, Faculdade de Medicina, Universidade de São Paulo (USP), São Paulo, SP, Brazil; Full Professor, Department of Cardiopulmonary Diseases, Faculdade de Medicina, Universidade de São Paulo (USP), São Paulo, SP, Brazil; Director of the Scientific Department, Associação Paulista de Medicina (APM), São Paulo, SP, Brazil.

Artificial intelligence (AI) has long fascinated humanity and has been vividly depicted in several movies, such as "2001: A Space Odyssey" with HAL 9000 and "The Terminator" with Skynet. The topic has gained even more traction in recent years, both in the media and everyday life with the advent of tools such as ChatGPT (Open AI), which was launched in November 2022. These AI applications are now utilized for a wide range of tasks, from seeking travel advice to generating intricate mathematical tables for economic studies.

In this brief editorial, we will explore an AI application that significantly impacts the quality and volume of both general and medical science: the use of AI in scientific writing. According to ChatGPT, scientific writing "is a writing style used to present research information and results clearly, concisely, and accurately, following specific rules and structures. It is essential for the dissemination of scientific knowledge, enabling researchers, professionals, and the general population to understand, evaluate, and replicate studies and experiments".^1^

As conducting scientific research and preparing data for publication are tasks that demand substantial time and effort, even for seasoned authors, AI tools have garnered significant interest from numerous researchers. In 2024, Weidman^2^ listed useful AI-based tools that are beneficial in various stages of the research process. These include tools for designing research questions (Elicit AI), identifying scientific databases (Search Smart), reviewing and analyzing the literature (Litmaps, Consensus, Connected Paper, ResearchRabbit, Scite, OpenRead), interpreting and synthetizing data (ChatGPT4, ResearchGPT, Lateral), structuring and writing academic papers and scientific articles for publication or for securing funding (Jenni.ai, Quillbot), translating texts into English (Google Translator, ChatGPT), and checking grammar (Grammarly).

AI tools can undoubtedly expedite various stages of scientific writing, including data analysis and statistics. They assist in recognizing data trends, providing contextual information, and enhancing linguistic accuracy, which is particularly crucial for non-native English-speaking authors. However, certain tasks still necessitate human intervention: reasoning, applying and integrating knowledge to address complex problems, demonstrating true creativity, and developing groundbreaking theories.^3^ The value of AI-generated outputs heavily relies on the breadth and quality of the sources powering these tools.^2^ Often, these sources lack transparency and exhibit inconsistent quality, both within a single tool and across tools, depending on how queries are formulated.

Authorship and plagiarism have also sparked considerable debate. As AI generates contents based on existing sources, and these sources may not be clearly referenced, the resulting material might be considered plagiarized.^4^ This raises the question of who should receive credit for AI-generated content: the person who created the question, the one who typed the prompt, the programmer, or the AI owner?

Furthermore, the way human authors interpret and use AI-generated content is a cause for concern. According to Anderson and Rainie, "considering the lack of user understanding of how these models derive their outputs, there are significant concerns about objectivity, bias, and fairness. This can lower the quality of academic work and oversimplify subtle academic arguments, ultimately leading to a loss of innovation and original critical thinking".^5^

In conclusion, AI tools can significantly assist the scientific process by enhancing the construction of scientific knowledge. However, their proper and human-supervised use, along with addressing referencing and plagiarism issues, remain critical concerns that need to be debated and regulated by the global scientific community.
